# Ras-p53 genomic cooperativity as a model to investigate mechanisms of innate immune regulation in gastrointestinal cancers

**DOI:** 10.18632/oncotarget.27983

**Published:** 2021-09-28

**Authors:** Austin R. Dosch, Walid K. Chatila, Yuguang Ban, Anna Bianchi, Nilesh U. Deshpande, Iago De Castro Silva, Nipun B. Merchant, Jashodeep Datta

**Affiliations:** ^1^Division of Surgical Oncology, Dewitt Daughtry Department of Surgery, University of Miami Miller School of Medicine, Miami, FL, USA; ^2^Sylvester Comprehensive Cancer Center, Miami, FL, USA; ^3^Center for Molecular Oncology, Memorial Sloan Kettering Cancer Center, New York, NY, USA; ^4^Department of Public Health Sciences; University of Miami Miller School of Medicine, Miami, FL, USA

**Keywords:** Ras-p53 cooperativity, Ras, TP53, immune, gastrointestinal cancer

## Abstract

Despite increasingly thorough mechanistic understanding of the dominant genetic drivers of gastrointestinal (GI) tumorigenesis (e.g., Ras/Raf, *TP53*, etc.), only a small proportion of these molecular alterations are therapeutically actionable. In an attempt to address this therapeutic impasse, our group has proposed an innovative *extreme outlier* model to identify novel cooperative molecular vulnerabilities in high-risk GI cancers which dictate prognosis, correlate with distinct patterns of metastasis, and define therapeutic sensitivity or resistance. Our model also proposes comprehensive investigation of their downstream transcriptomic, immunomic, metabolic, or upstream epigenomic cellular consequences to reveal novel therapeutic targets in previously “undruggable” tumors with high-risk genomic features. Leveraging this methodology, our and others’ data reveal that the genomic cooperativity between Ras and p53 alterations is not only prognostically relevant in GI malignancy, but may also represent the incipient molecular events that initiate and sustain innate immunoregulatory signaling networks within the GI tumor microenvironment, driving T-cell exclusion and therapeutic resistance in these cancers. As such, deciphering the unique transcriptional programs encoded by Ras-p53 cooperativity that promote innate immune trafficking and chronic inflammatory tumor-stromal-immune crosstalk may uncover immunologic vulnerabilities that could be exploited to develop novel therapeutic strategies for these difficult-to-treat malignancies.

## INTRODUCTION

The widespread availability and frequent inclusion of high throughput genomic sequencing technology in cancer medicine has enabled comprehensive characterization of the pan-cancer mutational landscape. Moreover, knowledge of the molecular underpinnings of tumorigenesis has accelerated drug discovery targeting dominant oncogenic drivers in specific cancers—ushering in the era of “precision oncology”—as well discovery of novel biomarkers that predict such drug responses and/or inform prognosis. These valuable insights into the “genotype-phenotype chasm”—i.e., how these molecular alterations dictate tumor biology—are particularly relevant in gastrointestinal (GI) malignancies, where outcomes with standard multimodality cancer treatment (e.g., surgery, chemotherapy, radiotherapy, etc.) continue to be dismal compared with many other solid cancers [1]. However, despite thorough mechanistic understanding of the dominant genetic drivers of GI tumorigenesis, including oncogenic Ras/Raf activation, deleterious *TP53* mutations resulting in p53 inactivation, loss of SMAD4, etc., only a small proportion of these molecular alterations are therapeutically actionable [2].

In an attempt to address this therapeutic impasse, our group has proposed an innovative model to decipher novel molecular vulnerabilities in high-risk GI cancers using an extreme outlier approach, highlighted in several recently published manuscripts. In this model, we aim to identify co-occurrent alterations between critical oncogenic drivers that are not only associated with extremes of oncologic outcome (i.e., survival, pathologic response, etc.) but also dictate prognosis [3, 4], correlate with distinct patterns of metastasis [3], and define therapeutic sensitivity or resistance [5]. Beyond simply identifying these cooperative molecular alterations that underlie high-risk clinical phenotypes in GI cancers, our model also proposes comprehensive investigation of their downstream transcriptomic, immunomic, metabolic, or upstream epigenomic cellular consequences [6]. We hypothesize that the iterative investigative journey from extreme outlier clinical phenotypes to identification of underlying high-risk genotypes to discovery of multi-omic repercussions of these alterations in GI malignancies will reveal novel therapeutic targets in previously “undruggable” tumors with high-risk genomic features.

**Figure 1:**
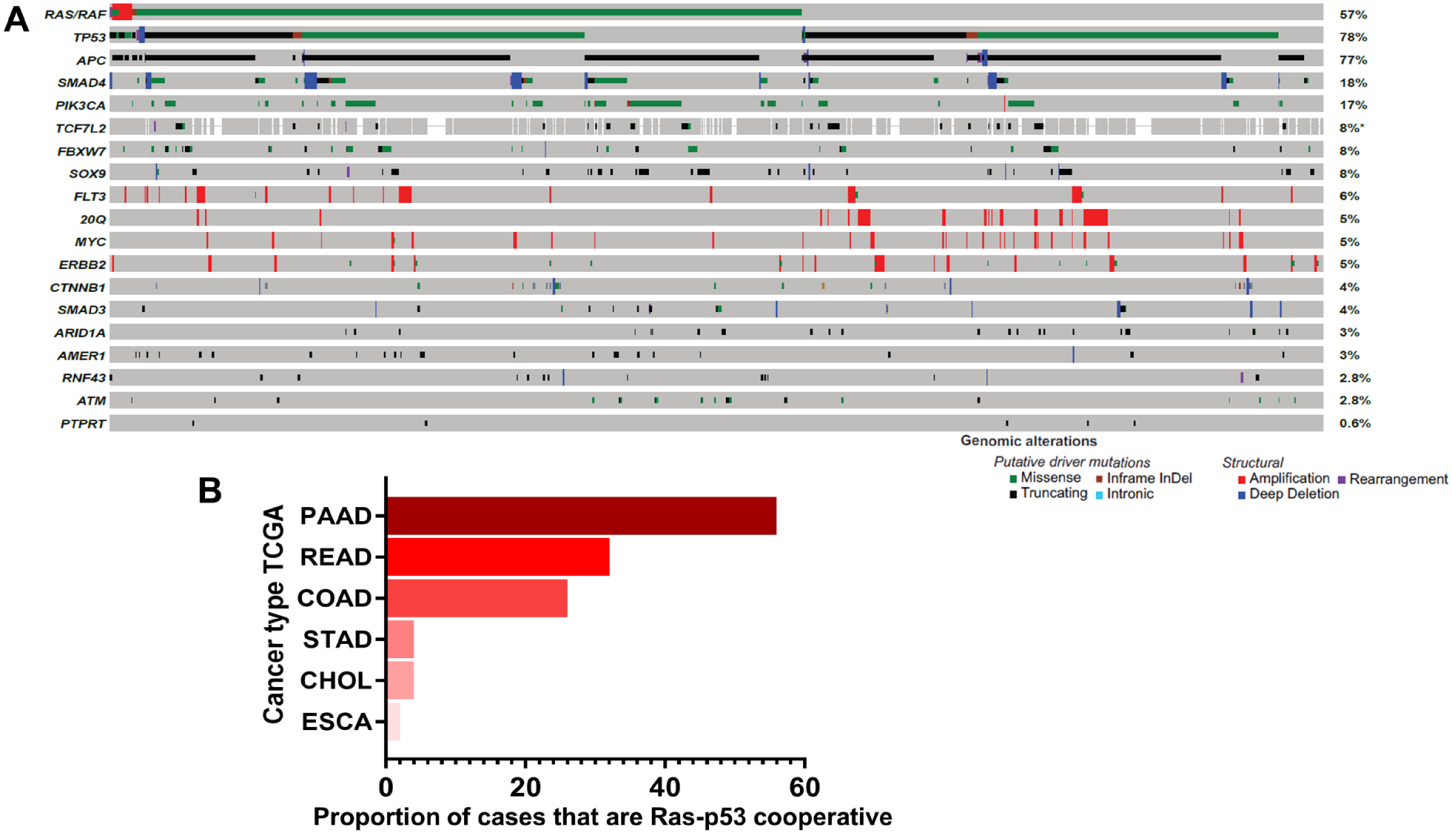
Ras-p53 genomic cooperativity in gastrointestinal cancer. (**A**) Oncoprint demonstrating most frequent putative driver alterations in patients with colorectal cancer in the MSK-IMPACT Clinical Sequencing Cohort (available through http://www.cbioportal.org). Gene names are provided to the left and mutational frequencies to the right. Genomic alterations are classified as putative driver or structural alterations in adjoining legend. (**B**) Frequency of co-altered Ras pathway and p53 alterations were determined in patients with pancreatic cancer (PAAD), rectal cancer (READ), colon cancer (COAD), stomach cancer (STAD), cholangiocarcinoma (CHOL), and esophageal (ESCA) cancer in The Cancer Genome Atlas (TCGA) dataset.

Leveraging this methodology, our group is actively pursuing the genomic cooperativity between Ras and p53 alterations in GI malignancy. In a recent manuscript in “Clinical Cancer Research”, we utilized an extremes-of-survivorship approach in patients with resected colorectal cancer liver metastasis (CRLM) and demonstrated that concurrent mutations in both *Ras* pathway and *TP53* alterations were significantly more frequent in ≤ 2-year survivors, whereas co-altered Ras-*TP53* was absent in ≥ 10-year survivors (67% vs. 0%, *P* < 0.001) [3]. In a separate manuscript recently published in “Annals of Surgery”, Ras-p53 cooperative mutations defined systemic and liver-directed chemotherapy resistance in patients with unresectable CRLM and was independently associated with worse survival (HR 2.52, 95% CI:1.37–4.64, *p* = 0.003) after controlling for conversion to surgical resection, liver metastasis burden, preoperative extrahepatic disease, and use of chemotherapy [5]. In yet another manuscript, currently in press [Narayan R, Datta J *et al*., personal communication], Ras-*TP53* cooperativity was associated with earlier local and distant recurrence in CRLM patients undergoing complete resection followed by adjuvant systemic and liver-directed chemotherapy. Not only is Ras-p53 genomic cooperativity oncologically significant, but also has broad clinical relevance by virtue of its frequent occurrence in colorectal cancers, with studies reporting concurrent Ras-p53 mutations occurring in nearly one-third of patients with colorectal liver metastasis [7]. Published data from the MSKCC database (publicly available through http://www.cbioportal.org) corroborate these statistics [8], with Ras-p53 cooperative alterations present in nearly a third of sequenced patients (Figure 1A). Collectively, these observations not only provide novel insight into the biologic relevance of Ras-p53 cooperativity in the clinical arena, but also establish Ras-p53 cooperativity as a high-risk genomic subgroup of GI cancers that manifest a clinical phenotype defined by chemoresistance, aggressive non-salvageable metastatic proclivity, and dismal cancer-related survival.

Data from The Cancer Genome Atlas (TCGA) also demonstrate that the RTK-Ras and p53 oncogenic pathways are co-altered in a substantial portion of patients with non-colorectal gastrointestinal cancers, particularly pancreatic ductal adenocarcinoma (PDAC) in which more than half of all patients demonstrate Ras-p53 cooperative alterations (Figure 1B). These data are particularly relevant because Ras-p53 cooperativity represents a foundational molecular event in GI tumorigenesis, modulating cellular signaling to induce the spontaneous development of invasive cancers in well-established genetically-engineered mouse models (GEMM) [9–11]. In-depth analysis in these sophisticated models suggest an interdependence on activating Ras pathway mutations and p53 loss in order to bypass cell-intrinsic mechanisms that abrogate tumor growth [12], and perhaps explain why mutations in these two specific pathways occur simultaneously in GI cancer patients. In addition, inactivating mutations in p53 not only abolish its ability to bind consensus DNA sequences and transactivate p53 target genes, but also enable the mutant p53 protein to acquire new oncogenic properties that are independent of wild-type p53 [13], further engendering Ras-p53 oncogenic cooperation. Beyond sufficiency to induce spontaneous tumorigenesis, Ras-p53 cooperativity also promotes invasion and motility and generates a highly metastatic phenotype *in vivo* [14, 15]. Recent insight into the molecular underpinnings of the pro-metastatic phenotype generated by Ras-p53 cooperativity revealed that oncogenic KRAS effectors activate CREB1 to allow physical interactions with mutant p53 that hyperactivate multiple pro-metastatic transcriptional networks. Moreover, mutant p53 and *CREB1* upregulate the pro-metastatic transcription factor *FOXA1*, while promoting Wnt/β-catenin signaling, together driving metastasis [16]. Finally, oncogenic cooperativity between Ras and p53 signaling is also mechanistically supported by the phenomenon of oncogene-induced senescence, whereby activating Ras pathway mutations induce a senescent program that results in replicative arrest of tumor cells [17]. Interestingly, this Ras-induced senescent program can be overcome by p53 inactivation (or p16 loss), leading to continued proliferation and escape from senescence-induced growth inhibition [18, 19]. Therefore, multiple lines of evidence underscore the critical role of Ras-p53 cooperativity in driving aggressive clinical phenotypes in GI cancer.

While the prognostic relevance of Ras-p53 genomic cooperativity and its mechanistic underpinnings are undoubtedly compelling, neither mutant Ras nor p53 are yet considered therapeutically actionable. As mentioned previously, we believe that deciphering how these cooperative driver mutations orchestrate tumor-promoting and immunosuppressive tumor-stromal-immune interactions in the tumor microenvironment (TME) to promote treatment resistance may reveal novel therapeutic opportunities for these aggressive cancers. As such, our group is particularly interested in understanding and targeting novel immune repercussions of Ras-p53 cooperativity in GI cancers. While the independent roles of both oncogenic Ras activation and p53 loss in establishing pro-inflammatory signaling and the recruitment and activation of immunosuppressive cells is well established [20–22], how Ras-p53 cooperativity encode unique tumor cell-intrinsic transcriptional programs to promote immunologic remodeling of the TME in gastrointestinal cancers is incompletely understood. Early insight into such coordinated tumor-intrinsic programs driven by Ras-p53 cooperativity came from an elegant study by McMurray and colleagues in which murine colon cells containing individual or combined mutants of p53 (p53^R175H^) or activated H-Ras (Ras^G12V^) were profiled by microarray analysis to delineate a set of gene transcripts—encompassing a broad range of functional annotations such as signal transduction, metabolism, cell adhesion, etc.—that are synergistically regulated by Ras-p53 cooperative signaling [23]. Further investigation demonstrated that a majority of these genes are critical for tumor development, as evident by attenuation of tumor growth in mice following gene perturbation experiments.

**Figure 2:**
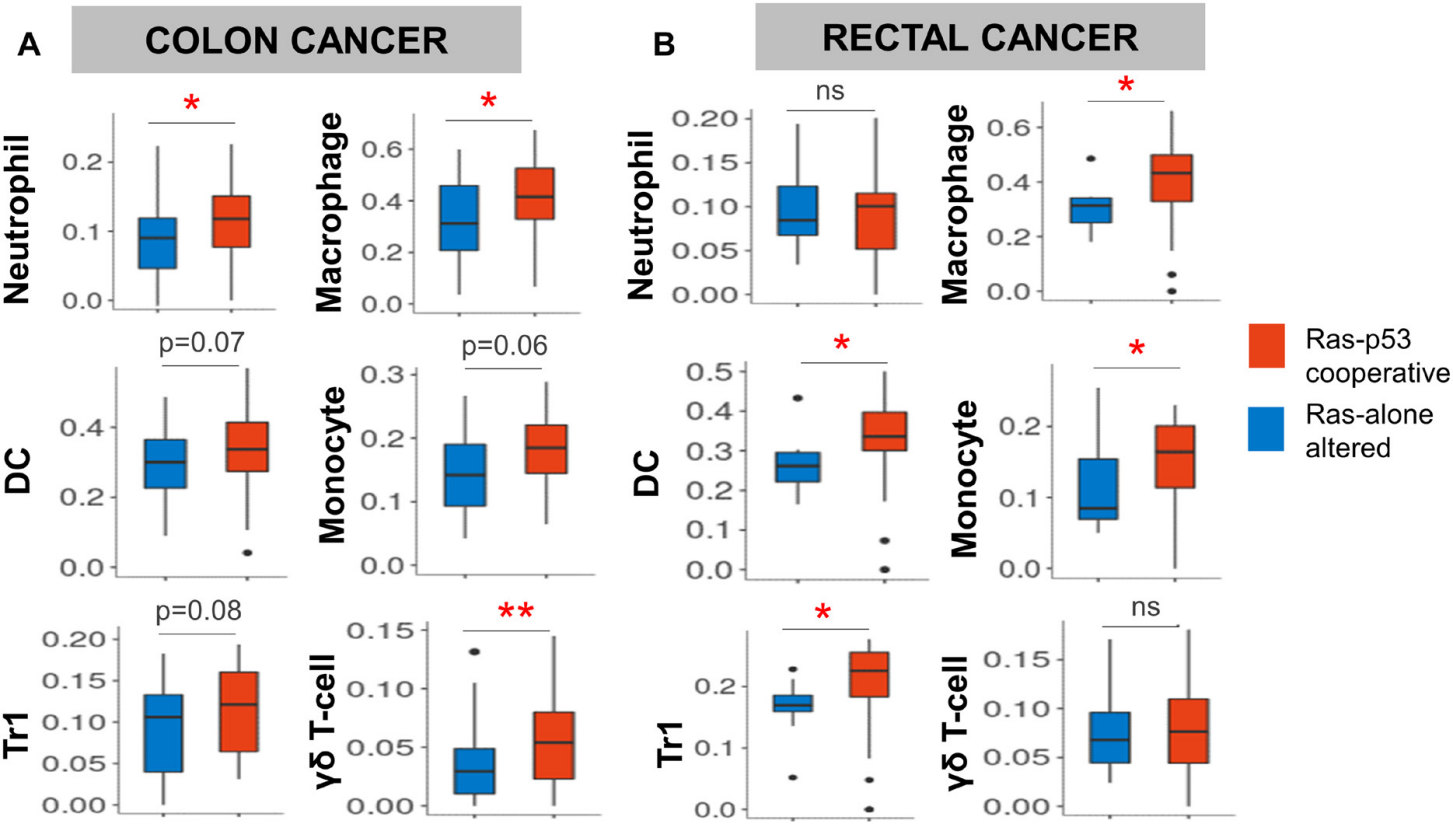
Ras-p53 cooperative mutations in rectal and colon cancer are associated with an innate immunoregulatory phenotype. Single-cell immune deconvolution from bulk RNA sequencing data from colon and rectal cancer cases curated from The Cancer Genome Atlas (TCGA) Pan-Cancer database was performed using *ImmuneCellAI* as described in Materials and Methods. (**A** and **B**) Comparison of neutrophil, macrophage, dendritic cell (DC), monocyte, inducible T regulatory type 1 (Tr1), and γδ T-cell signatures between Ras-alone (blue) and Ras-p53 co-operative (red) (A) colon and (B) rectal cancer samples in the TCGA database. ns, not significant; ^*^ *p* < 0.05; ^**^ *p* < 0.01.

In a complementary study, Buganim and colleagues utilized an *in vitro* transformation model using immortalized human fibroblasts transduced with Ras^G12V^ and p53^R175H^ constructs to identify an inflammation-associated gene signature synergistically induced by cooperative Ras-p53 alterations [24]. Among these were several genes related to innate immune cell recruitment and activation such as ELR chemokines (i.e., *CXCL1, CXCL2, CXCL3,* and *CXCL6)* and well as the pro-inflammatory ligands (i.e., *IL1B, IL6, IL8,* and *CSF2*) (Table 1). To explore these intriguing data further, we performed an analysis of whole tumor transcriptomic data from the colon (COAD) and rectal cancer (READ) datasets of the TCGA. Using established single-cell immune deconvolution pipelines (ImmuneCellAI [25]), we compared the computationally inferred immune populations in primary COAD and READ tumors harboring concurrent hotspot alterations in Ras (i.e., *KRAS*, *NRAS*, or *BRAF*) and p53 versus tumors with Ras alterations alone. Ras-p53 cooperative COAD/READ tumors exhibited increases in immunosuppressive innate immune populations such as tumor-associated neutrophils, tumor-associated macrophages (TAMs), monocytes, inducible T-regulatory type 1 (Tr1) cells, and γδ T-cells (Figure 2A and 2B). Recent findings from a study by Blagih and colleagues expand on these hypothesis-generating data in *in vivo* models of GI cancer to indicate an association between cooperative Ras-p53 alterations and the recruitment of innate immune populations into established tumors. In this study, concurrent *KRAS* and *TP53* mutations coordinated the influx of myeloid cells into tumors, but did not affect adaptive immune subsets such as regulatory T-cells. Using an inducible *KRAS^G12D/+^* construct in PDAC tumor cells containing biallelic loss of *TP53*, the authors demonstrated that withdrawal of *KRAS* mutations resulted in a significant reduction in the levels of intratumoral CD11b^+^ myeloid cells, CD11b^+^F4/80^+^ macrophages, and CD11b^+^CXCR3^+^ tumor-associated myeloid cells, highlighting the importance of cooperative Ras-p53 mutations in driving innate immune cell recruitment in pancreatic cancer [26]. These findings strongly link concurrent Ras-p53 mutations with the establishment of innate immunoregulatory signaling within GI tumors and may represent the molecular events initiating the recruitment and activation of innate immune cells that sustain immunosuppression within the GI cancer TME.

**Table 1:** Differentially overexpressed transcripts associated with innate immunity induced by cooperative Ras-p53 mutations

ELR Chemokines
*CXCL1*
*CXCL2*
*CXCL3*
*CXCL6*
**Pro-Inflammatory Ligands**
*IL1B*
*IL6*
*IL8*
*CSF2*
**Extracellular Matrix-related Genes**
*MMP3*
*CLECSF2*

Data from Buganim et al. demonstrating increased expression of selected chemokines (i.e., *CXCL1*, *CXCL2*, *CXCL3*, and *CXCL6*) and pro-inflammatory ligands (i.e., *IL1B*, *IL6*, *IL8*, and *CSF2*) from immortalized human fibroblasts transduced with Ras^G12V^ and p53^R175H^ mutant constructs [24].

Taken together, prior and emerging data indicate that Ras-p53 genomic cooperativity is an ideal model to investigate mechanisms of innate immune regulation in gastrointestinal cancers. Innate immune populations such as neutrophils, neutrophilic myeloid-derived suppressor cells (MDSCs), and TAMs are not only critically important for the initiation and progression of solid organ cancers [27], but have also been extensively implicated in dictating responses to chemotherapy and/or immunotherapy in GI cancers, making efforts to further understand and characterize the mechanisms governing their influx into and function within the TME critical to improving patient outcomes [28–31]. As such, while is appears clear that Ras-p53 cooperativity is associated with innate immune trafficking into the GI TME, the specific transcriptional programs encoded by Ras-p53 cooperativity that govern innate immunoregulation in the TME have not been extensively explored. Ongoing efforts by our group are focused on comprehensively understanding the diverse transcriptional programs encoded by cooperative Ras-p53 alterations in GI cancers that promote chronic inflammatory tumor-stromal-immune crosstalk, innate immune trafficking, immune exclusion, and therapeutic resistance. We believe that these investigations will uncover novel immunologic vulnerabilities that could be exploited to develop therapeutic strategies to mitigate these immunomodulatory effects, overcome immune exclusion and therapeutic resistance, unleash anti-tumor immunity, and ultimately revolutionize treatment for patients with these difficult-to-treat GI cancers.

## MATERIALS AND METHODS

Previously published genomic data [3, 8] was curated from http://www.cbioportal.org [32, 33] and depicted using an oncoprint to visualize the frequency of driver, putative driver, and variants of undetermined significance mutations in all patients with colorectal cancer (*n* = 935) at MSKCC that had undergone sequencing using the MSK-IMPACT platform.

The TCGA pan-cancer database was queried for all hotspot mutations in Ras family members (*KRAS*, *NRAS*, *HRAS*) and *TP53*, and the frequency of co-altered Ras-*TP53* mutations across selected GI cancers was tabulated. TCGA samples in the COAD and READ datasets were dichotomized into Ras-p53 cooperative and Ras-alone altered, and resulting fragments per kilobase of transcript per million (FPKM) reads from these two groups were entered into a publicly available single cell immune deconvolution pipeline ImmuneCellAI (available at: http://bioinfo.life.hust.edu.cn/ImmuCellAI#!/) [25]. ImmuneCellAI estimates the abundance of 24 immune cells from gene expression datasets, comprising 18 T-cell subtypes and 6 other immune cells: B-cell, NK cells, Monocyte, Macrophage, Neutrophil, and DC. Results from selected immune sub-populations in both COAD and READ datasets were depicted as box-and-whiskers plots.
